# Biochar Supported Nanoscale Iron Particles for the Efficient Removal of Methyl Orange Dye in Aqueous Solutions

**DOI:** 10.1371/journal.pone.0132067

**Published:** 2015-07-23

**Authors:** Lu Han, Song Xue, Shichen Zhao, Jingchun Yan, Linbo Qian, Mengfang Chen

**Affiliations:** 1 Key Laboratory of Soil Environment and Pollution Remediation, Institute of Soil Science, Chinese Academy of Sciences, Nanjing, 210008, China; 2 College of Environmental Science and Engineering, Suzhou University of Science and Technology, Suzhou, 215011, China; 3 College of Resource and Environmental Sciences, Nanjing Agricultural University, Nanjing, 210095, China; Peking University, CHINA

## Abstract

The presence of organic contaminants in industrial effluents is an environmental concern of increasing global importance. One innovative technology for treating contaminated industrial effluents is nanoscale zero-valent iron supported on biochar (nZVI/BC). Based on Transmission Electron Microscopy, X-Ray Diffraction, and Brunauer-Emmett-Teller characterizations, the nZVI was well dispersed on the biochar and aggregation was dramatically reduced. Methyl orange (MO) served as the representative organic contaminant for verifying the effectiveness of the composite. Using decolorization efficiency as an indicator of treatment effectiveness, increasing doses of nZVI/BC yielded progressively better results with 98.51% of MO decolorized by 0.6 g/L of composite at an nZVI/BC mass ratio of 1:5. The superior decolorization efficiency of the nZVI/BC was attributed to the increase in the dispersion and reactivity of nZVI while biochar increasing the contact area with contaminant and the adsorption of composites. Additionally, the buffering function of acid-washed biochar could be in favor of maintaining the reactivity of nZVI. Furthermore, the aging nZVI/BC for 30 day was able to maintain the removal efficiency indicating that the oxidation of nZVI may be delayed in the presence of biochar. Therefore, the composite of nZVI/BC could represent an effective functional material for treating wastewater containing organic dyes in the future.

## Introduction

The discharge of dye-containing wastewaters have long represented a major environmental challenge because of aesthetic concerns, their potential toxicity, and carbonaceous oxidation demand on receiving waters. Dye wastewaters are characterized by more complicated and stable structure, tending to be quite persistent in the environment and resistant to biodegradation [1,2]. It is estimated that more than 70,000 tons of dye wastewaters are discharged worldwide every year [3]. Various attempts have now been made for treatment of organic dyes from industrial effluents. In particular, chemical decomposition has been quite an effective method for relieving the environmental pressure from organic dyes-containing effluents. It is well recognized that nZVI, as an environmentally benign chemical reductant, is capable of effectively transforming and immobilizing a wide range of contaminants including halogenated compounds [1,4,5], heavy metals [6,7], nitrate [8], dye and textile compounds [9,10]. However, despite its significantly high reactivity surface energy, the intrinsic ferromagnetic property of nZVI can cause severe aggregation resulting in a loss of reactivity, durability and mechanically strength [11]. In order to overcome the disadvantage, efforts have been made to modify nZVI to optimize the dispersion and/or stabilization of nZVI. Some mechanical stable supports were proposed such as zeolite [12], resin [13], clay mineral [14], graphene [15] and active carbon [16,17] to disperse the iron particle sizes. In particular, clay mineral and porous carbon are two commonly used supporting materials for nZVI. However, clay minerals usually have lower specific surface area relative to nZVI, therefore, the specific surface area of composite will be lower than that of iron nanoparticles [18,19]. In comparison with clay minerals, biochar is a porous carbon-rich material, which is characterized by a higher specific surface area, greater adsorption capacity, and stable structure. Due to the porous structure of biochar, functional nanoparticles based on biochar for environmental application has recently been successfully synthesized, and demonstrated to have excellent abilities to remove a range of contaminants from aqueous solutions [20,21]. Biochar can be derived from a variety of biomass such as wood, leaves, manure and agricultural wastes through the pyrolysis under oxygen limited conditions [22]. In addition, it does not need a treatment for further activation like active carbon [23]. Therefore, biochar may be a more cost-effective and readily accessible alternative material for engineering application as a template of nZVI. Extensive attention has been paid to biochar that sequestrates numerous organic and inorganic contaminants and it is considered as a significant material in the environmental remediation [24–26]. Therefore, it is considered necessary to evaluate the effectiveness of biochar ameliorating the aggregation of iron nanoparticles and dual mechanisms of biochar supporting nZVI in adsorbing and degrading organic pollutants.

MO was used as a representative dye to evaluate the removal effect by the modified iron nanoparticles in the present study. As a simple model of a series of common azo dyes, MO is an anionic dye and widely used in chemical, textile and paper industries. Its structure is characterized by a mono-azo bond and sulphonic group, which are responsible for its visible color and the high solubility in water, respectively. The MO molecules may be fixed in biochar by sorption or be transformed to simple, low molecular weight products by nZVI through the destruction of the azo bond N = N. Both of the two processes will lead to the decolorization of MO, therefore, the decolorization was chosen as the primary indicator of technology effectiveness. The objectives of the study are to 1) synthesize and characterize the biochar supported nZVI composite material; 2) verify the effectness of prepared nZVI/BC composite for MO decoloration and 3) to explore the MO decolorized mechanism by nZVI/BC.

## Experimental

### Materials

Potassium borohydride (KBH_4_) (>98%) was purchased from Sinopharm Chemical Reagent Co., Ferrous sulfate (FeSO_4_·7H_2_O) (99.7%) was obtained from Guangdong Chemical Reagent Co., MO (C_14_H_14_N_3_SO_3_Na) was from Beijing Chemical Reagent Co., Rice husk-derived biochar (BC) was supplied by Nanjing Zhongheng Co. prepared at 500 °C. All other reagents were analytical reagent grade. Deionized (DI) water was used throughout this study.

### Particle Preparation

Biochar (<100-mesh) was firstly mixed with 1 M HCl (1/20, v/v) and shaken overnight at room temperature for demineralization, such as K^+^, Na^+^, Ca^2+^ and Mg^2+^. Then biochar was then purified using dialysis method until the solution pH was close to neutral and dried at 80 °C in an oven.

For the synthesis of biochar supported nZVI (nZVI/BC), an aqueous phase reduction of ferrous iron (FeSO_4_•7H_2_O) was applied with the reported method [27] using potassium borohydride (KBH_4_) as a reductant. All solvents were degassed and saturated with N_2_ for more than 60 min prior to use and the whole synthesis process was under N_2_ atmosphere. The freshly prepared FeSO_4_·7H_2_O (0.05 M) in methanol/deionized water (3/7, v/v) solution was mixed with a measured mass of BC stirring for 60 min to form a homogenous solution. An equal volume of 0.1 M KBH_4_ solution was added drop wise into the slurry under vigorous stirring. Following the complete reaction, the composite sample was separated from the supernatant and biochar unsupported zero iron with a magnet. Subsequently, the obtained sample was washed and purified by deionized water and methanol for several times, respectively. nZVI/BC composites with different theoretical mass ratios of nZVI/BC at 1:3, 1:5, and 1:7 (referred hereafter to as nZVI/BC_3_, nZVI/BC_5_, nZVI/BC_7_, respectively) were prepared. Unsupported nZVI was also prepared according to the method mentioned above without biochar being added. The nZVI and nZVI/BC particles were stored in ethanol under N_2_ atmosphere and vacuum-dried prior to use.

### Characterization of nZVI/BC

The iron particle sizes and morphology were analyzed using a Hitachi HT7700 (Hitach, Japan) Transmission Electron Microscope (TEM) at an operating voltage of 100 kV. Possible sample crystal structures of nZVI, nZVI/BC, and biochar were performed on a Thermo ARL X'TRA X-ray diffratometer (Thermo Fisher Scientific, USA) with Cu Kα X-ray source at 40kV and 40mA. The N_2_-Brunauer-Emmett-Teller (BET) specific surface area and total pore volume of particles were measured using an ASAP 2020 BET-surface area analyzer (Micromeritics, USA).

### Batch experiments

A 1,000 mg/L aqueous MO stock solution was prepared and used to create serial dilutions for the batch experiments. The experiments were conducted in triplicate under anaerobic conditions.

To realize the highest efficiency of iron nanoparticles in nZVI/BC composites decolorizing MO, three different mass ratios of nZVI/BC (1:3, 1:5, or 1:7) were used to decolorize the 200 mL of 60 mg/L MO solution at ambient temperature with the initial pH uncontrolled, respectively, and the content of Fe^0^ was maintained at 0.1 g/L in each composite. 5 mL solution was withdrawn at regular intervals, filtered through 0.22 μm membrane filter, and the concentrations were determined by UV-visible spectrophotometer (UV-2102PCS, Shanghai, China) at the maximum absorption intensity of 464 nm (diluted if necessary before measured) [28]. Individual biochar and nZVI were also added to MO solution as control experiments. The pH was determined by a digital pH meter (pHS-3C, Shanghai, China).

Various factors for the removal of MO by nZVI/BC were investigated: (1) the starting concentrations of MO ranging from 200 to 600 mg/L with the nZVI/BC_5_ dosage of 0.6 g/L; (2) the nZVI/BC loading being increased from 0.3 to 0.9 g/L decolorizing 300 mg/L of MO; (3) the initial solution pH being adjusted with 0.1 M HCl or 0.1 M NaOH solution to 4.06 to 9.13, and (4) coexistent anions including Cl^-^, SO_4_^2-^, and NO_3_^-^ (10-fold molar concentration of MO).

To evaluate the chemical corrosion of the prepared materials, nZVI/BC_5_ and nZVI were kept in a capped glass bottle under natural air atmosphere for a month, respectively. Individual removal of the initial 300 mg/L MO solution by aging nZVI/BC_5_ (0.6 g/L) and nZVI (0.1 g/L) were undertaken. In comparison with the aging materials, respective removal of MO by freshly prepared nZVI/BC_5_ and nZVI was also undertaken under the same conditions.

The decolorization efficiency (η%) of MO and the unit removal capacity (mg/g) were calculated using the following Eqs (1) and (2):

$$\eta =\frac{\left(C_{0}-C_{e}\right)}{C_{0}}\times 100\text{\%}$$ (1)

$$\text{Unit removal capacity}=\frac{\left(C_{0}-C_{e}\right)}{m}$$ (2)

Where *C*_*0*_ and *C*_*e*_ are the initial and equilibrium MO concentrations (mg/L) in the solution respectively, *m* is the amount of nZVI/BC (g/L).

Non-linear regressions with empirical equation (first order exponential decay) was introduced according to the Eq (3) due to the residual MO concentration being taken into account [29].

$$C_{t}=C_{e}+\left(C_{0}-C_{e}\right){\text{*α*e}}^{-kt}$$ (3)

Where *C*_*t*_ is the concentration of MO solution (mg/L) at time *t* (min), *k* is the observed rate constant (min^-1^), α denotes the variation coefficient for each test.

## Results and Discussion

### Particle Characterization

The BET surface area (SA) and total pore volume of particles are presented in Table 1. The SA of nZVI was 20.89 m^2^/g and the SA of composite was significantly increased by introducing biochar (205.35 m^2^/g). The SA for nZVI/BC at various mass ratios of 1:3, 1:5, and 1:7 were 26.62, 142.80, 138.07 m^2^/g, respectively. It appears that more content of biochar in the composite of nZVI/BC was favorable for the dispersion of iron nanoparticles because the SA of nZVI/BC_5_ and nZVI/BC_7_ were much higher than that of nZVI/BC_3_. Nevertheless, nZVI/BC_7_ slightly decreased the SA compared with nZVI/BC_5_. This is because that nZVI nanoparticles were synthesized among the layers of BC, therefore, higher biochar mass was favorable for the dispersion of nZVI on BC surface. However, further excess of biochar (nZVI/BC mass ratio 1:7 in the case) resulted in the increased aggregation of biochar sheets, and thereafter lower SA value was observed instead of improving the specific area [30].

**Table 1:** The Barrett-Emmett-Teller (BET) specific surface area and pore structural properties of particles.

Sample	BET-SA (m^2^/g)	Micropore volume (cm^3^/g)	Pore volume (cm^3^/g)	Pore size (nm)
BC	205.35	0.073	0.11	2.17
nZVI	20.89	0.00053	0.0072	10.00
nZVI/BC_3_	26.62	0.0041	0.033	4.99
nZVI/BC_5_	142.80	0.052	0.079	2.21
nZVI/BC_7_	138.07	0.047	0.081	2.34

**Fig 1:**
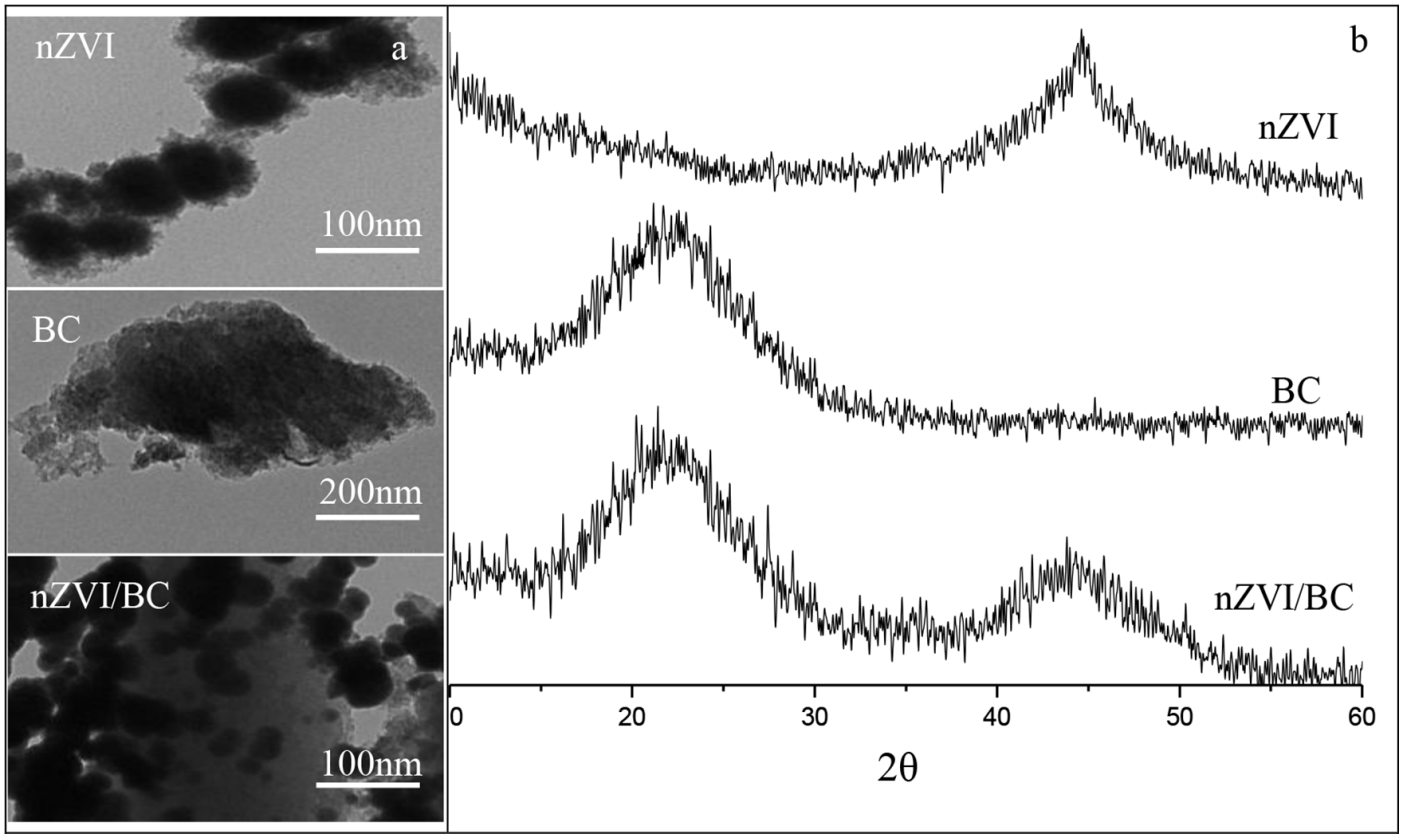
(a) Transmission Electron Microscope and (b) X-Ray Diffraction images of nZVI, BC and nZVI/BC.

The TEM images of nZVI, biochar, and nZVI/BC_5_ are presented in Fig 1a. The morphology of nZVI was characterized by spherical shape with particle sizes ranging from 50 nm to 100 nm, which was similar to that reported in the literature [31]. nZVI existed in the form of prominent chain-like aggregates because of their huge interface energy and magnetic property. Biochar was in amorphous flake shapes with the size of 300~500 nm and nZVI nanoparticles appeared to be dispersed on the surface and the boundary sites of biochar under the TEM images of nZVI/BC. Thus, it is expected that the dispersion and surface reactivity of nZVI was effectively improved using the biochar as templates of nZVI through the TEM and BET analyses [32,33].

The outcome of XRD analysis is provided in Fig 1b in order to reveal the possible crystal structures of the three materials. It is observed that α-Fe^0^ was confirmed on the basis of a broad peak centered at 44–45° 2θ [34] and XRD peak at 2θ = 20–25° was assigned to the amorphous structure of graphite of biochar [35]. Both characteristic diffraction peaks of Fe^0^ and biochar in the XRD spectrogram of nZVI/BC indicated successful synthesis of the composites.

### Effects of different mass ratios of nZVI/BC on MO decolorization

**Fig 2:**
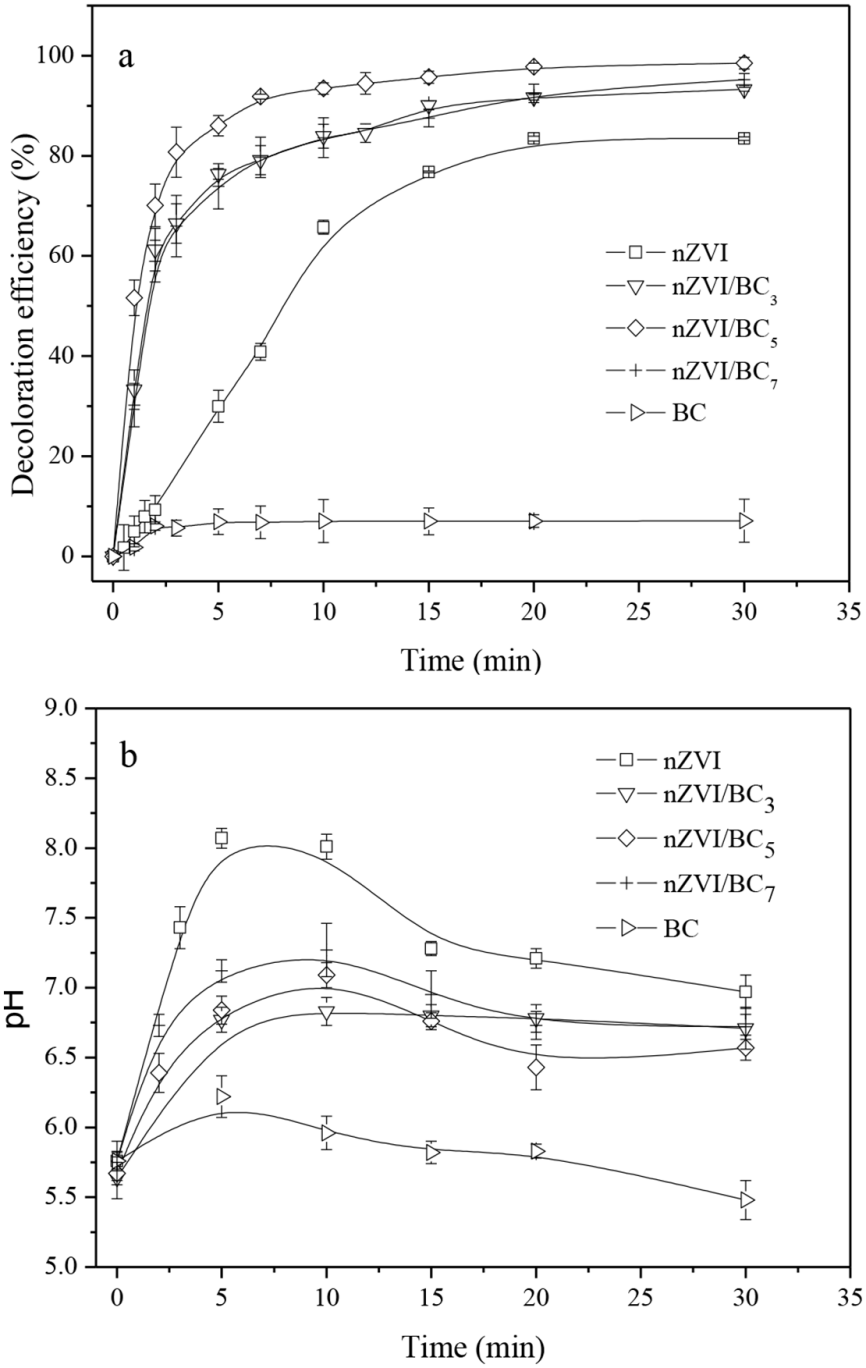
(a) Removal efficiencies of MO using different materials. (b) Changes of pH versus reaction time profiles. Conditions: In all cases, initial MO concentration = 60 mg/L, nZVI dosage = 0.1 g/L, the nZVI/BC mass ratio was 1:3, 1:5, and 1:7 for nZVI/BC_3_, nZVI/BC_5_, and nZVI/BC_7_, respectively; biochar dosage = 0.5g/L.

The effects of different mass ratios of nZVI/BC (1:3, 1:5, and 1:7) on MO decolorization are illustrated in Fig 2a. The decolorization efficiency of 83.4% was achieved for nZVI (0.1 g/L), while 0.5 g/L of biochar within the 30 min reaction time only adsorbed 7.1% of the initial MO concentration. Removal of dye contaminants by biochar usually depends on higher solid-liquid ratio of biochar and longer reaction time [1,36]. Thus, the biochar had relative low removal efficiency for MO in the experiment. By comparison, nZVI/BC significantly enhanced the removal efficiency of MO. The decolorization efficiencies were increased to 93.3%, 98.5%, and 95.2% for nZVI/BC_3_, nZVI/BC_5_, and nZVI/BC_7_ respectively. Both removal efficiencies and reaction rates of MO for nZVI/BC were all much higher than that of the nZVI containing the equal mass of Fe^0^ (0.1 g/L). In particular, approximately 93% of MO was decolorized by nZVI/BC_5_ in the first 7 min. The outcome for nZVI/BC_5_ was approximately 53% higher than that of nZVI and 10% higher than those of the nZVI/BC_3_ and nZVI/BC_7_. These results indicate that the iron nanoparticles on the biochar surfaces played important role in the enhanced removal of MO contaminant. MO is an anionic dye, which can be attracted to the surface of the positively charged nZVI particles because of the zero point of charge of the nZVI (pH_zpc_ ≈ 8) higher than that of the original pH of the solution (pH = 5.76) [37]. Furthermore, the Fe^0^ can destroy the chromophoric group of MO (-N = N-) into the colorless products. In addition, the nZVI/BC composites could serve as more adsorption sites for the MO due to the greater surface area than nZVI and the nZVI dispersion on biochar increased more reactive sites to react with the MO molecules. Therefore, the nZVI/BC composite could strongly remove the MO dyes from the aqueous solution [35,38].

It should be noted that higher amounts of biochar in nZVI/BC appeared to be more beneficial for the decolorization performance. The similar results were also reported by other literatures relative to clay minerals as carriers [38, 39]. However, excess biochar supported nZVI did not appear to give a more synergistic effect towards the removal of MO, as the removal efficiency of MO with nZVI/BC_7_ was slightly decreased by approximately 3% relative to nZVI/BC_5_. Deng proposed that the reactive sites responsible for reduction and the non-reactive sites responsible for adsorption were both on the iron surfaces [40]. Therefore, it is deduced that excess biochar loading may block the reactive sites on the iron surfaces leading to the decrease of the reducing power of the iron. As a result, a mass ratio of Fe/BC at 1:5 gave the optimum performance in the present experiment.

**Fig 3:**
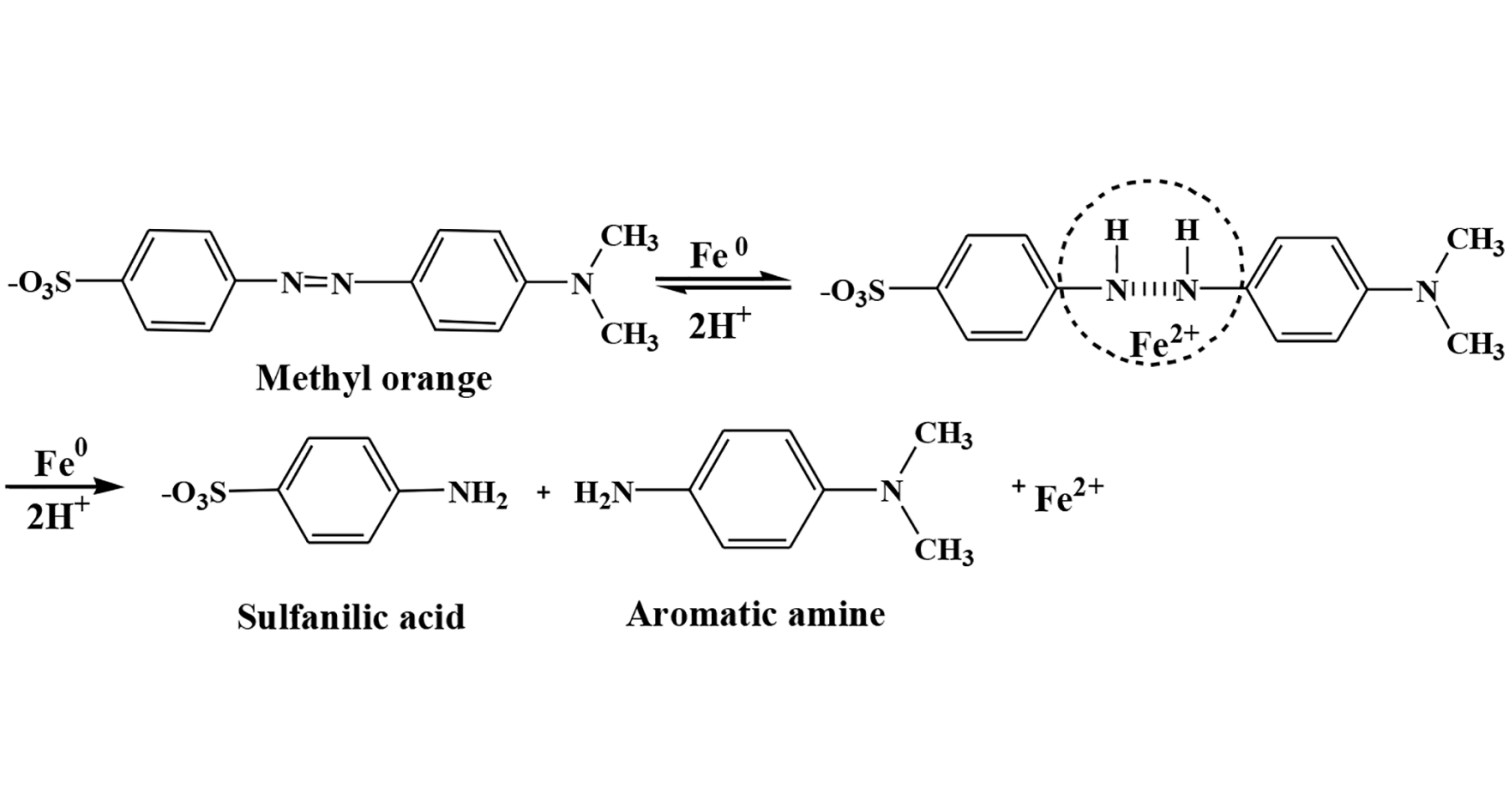
Degradation mechanism of methyl orange by Fe^0^.

Solution chemistry measurements suggested that the removal of MO was accompanied by a concurrent pH rise both in nZVI-MO and nZVI/BC-MO systems in the first 10 min (Fig 2b). This is because that Fe^0^ cleaved the azo N = N bond to form hydrazine-like N-H single bonds that would consume H^+^ and lead to pH increasing, as shown in Fig 3. Besides, the spontaneous reaction that Fe^0^ reacting with water to generate Fe^2+^, H_2_ and OH^-^ was also responsible for the increase of solution pH, as shown in the Eq (4) [41]. In the nZVI-MO system, pH fluctuated from 5.76 to 8.07 within 10 min. Subsequently, pH fell back to the vicinity of 7 in the last 20 min. This was ascribed to the consumption of H^+^ resulting in the alkaline condition normally favorable for Fe^2+^ reacting with OH^-^ in solution to form Fe(OH)_2_, as shown in the Eq (5) [31]. In comparison with nZVI, the buffering function of biochar in nZVI/BC-MO solutions maintained the pH ranging from 5.76 to average of 7.0, because a large amount of hydrogen ions were adsorbed to the biochar in acid solution rinsing process before the synthesis. They could well play a role of buffering solution pH and maintain the relatively high reactivity of nZVI. This could also be one of the factors enhancing the decolorization efficiency of MO.

$${\text{Fe}}_{\left(s\right)}^{0}+2{\text{H}}_{2}{\text{O}}_{\left(aq\right)}\to {\text{Fe}}_{\left(aq\right)}^{2+}+{\text{H}}_{2\left(\text{g}\right)}^{}+2{\text{OH}}_{\left(aq\right)}^{-}$$ (4)

$${\text{Fe}}_{\left(aq\right)}^{2+}+2{\text{OH}}_{\left(aq\right)}^{-}\to \text{Fe}{\left(\text{OH}\right)}_{2(s)}^{}↓$$ (5)

The observed kinetic constant was fitted according to the Eq (3) and the related parameters are presented in S1 Table. The results showed that the observed kinetic constant (k) of nZVI was 0.104 min^-1^. The k for nZVI/BC ranged from 0.476 to 0.743 min^-1^ at different mass ratios (1:3, 1:5, and 1:7), increasing by a factor of 4.58 to 7.13 compared with the nZVI. These suggest an excellent reducing capability of the nZVI/BC composite.

### Factors affecting removal of MO

A series of batch experiments were carried out to investigate the factors influencing the removal efficiency of MO such as the initial concentrations of MO, the nZVI/BC_5_ dosages, starting pH, and various anions.

**Fig 4:**
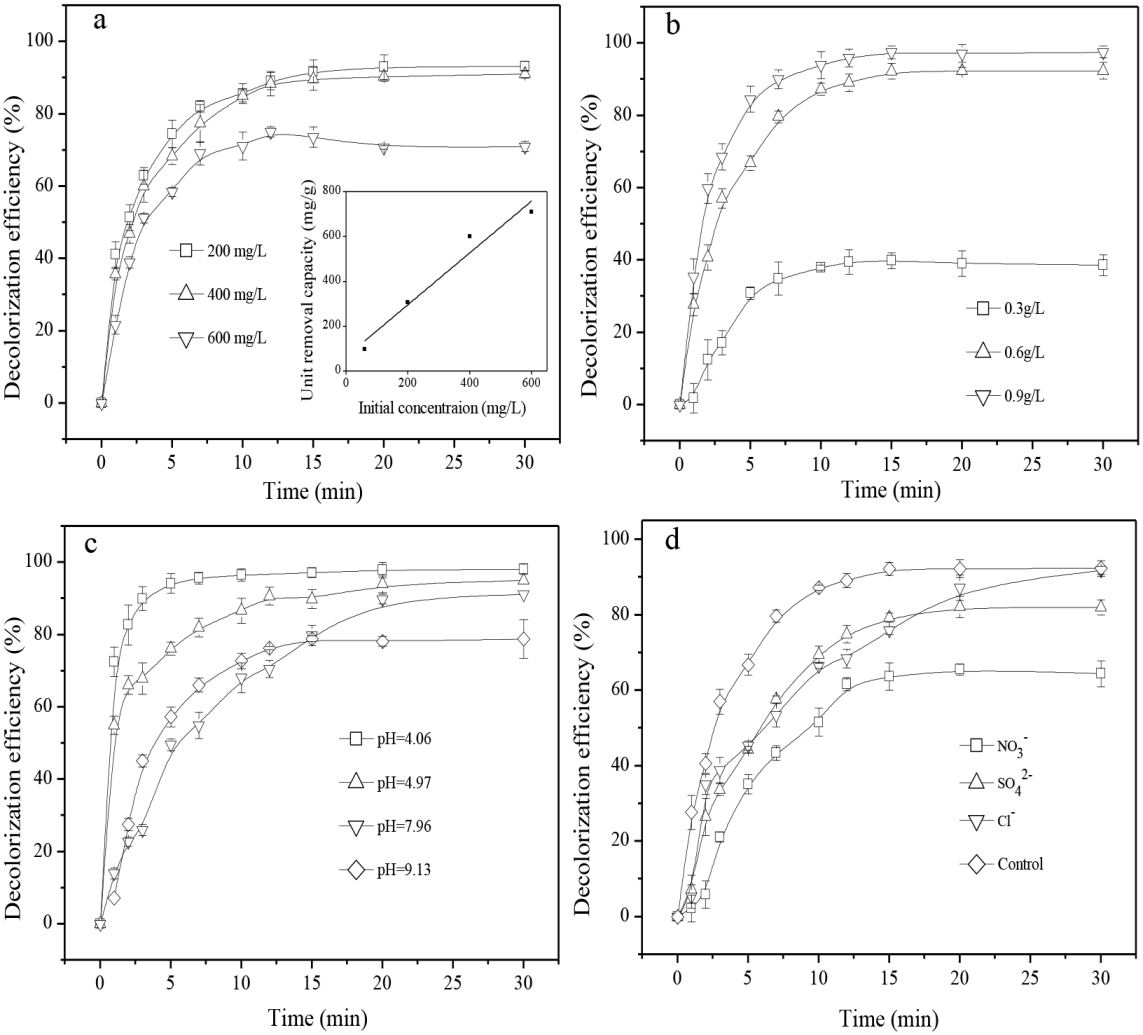
Factors affecting removal of MO: (a) different initial concentrations. The inset was the unit removal capacity versus different initial concentrations; (b) various nZVI/BC_5_ dosages; (c) various pH; and (d) various anions (10-fold molar concentration of MO).

The effects of initial dye concentrations are shown in Fig 4a. An average of 92.1% of removal efficiency was achieved for initial dye concentrations between 200 and 400 mg/L. However, it was decreased by 21.2% when the initial concentration reached 600 mg/L. Additionally, the apparent rate constants were decreased from 0.470 to 0.303 min^-1^ (S1 Table). However, the performances were superior to that of other similar supported or unsupported iron nanoparticles [30,42,43]. Furthermore, the unit removal capacity versus different initial concentrations were plotted proportionally a line (the inset in Fig 4a) with the linear coefficient (R^2^) of 0.96. The unit removal capacity was 97.8, 306.7, 605.0, and 709.1 mg MO per gram nZVI/BC_5_ for initial concentrations of 60, 200, 400, and 600 mg/L, respectively. This result was slightly lower than that of bentonite supported Fe/Pd (B/nZVI/Pd) nanoparticles [42]. However, 50% of Fe^0^ in 0.5 g/L B/nZVI/Pd loading in that literature increased the amount of Fe^0^ used in the present test by a factor of 2.5. In addition, the B/nZVI/Pd contained a catalyst, palladium, which could significantly enhance the removal efficiency. Thus, from the cost-effective point of view, nZVI/BC_5_ has a more significant superiority in practical applications and biochar-based nZVI may lower the overall treatment cost for dye-contaminated water.

The effect of nZVI/BC_5_ dosage on MO decolorization efficiency is shown in Fig 4b. When 0.3 g/L of nZVI/BC_5_ was loaded in solution, only 38.6% of MO was decolorized in the first 15 min and was quenched primarily in the last 15 minutes of the experiment. In comparison to that, when nZVI/BC_5_ dosage was increased from 0.6 to 0.9 g/L, the decolorization efficiency was increased from 92.1% to 97.3%. The observed rate constants ranged from 0.201 to 0.369 min^-1^ (S1 Table). It is clear that the more nZVI/BC_5_ were loaded, the more reactive surface sites were utilized by MO. Moreover, the collision between zero-valent iron atoms and MO molecules became more intense, leading to improve the reaction rates.

The effects of different initial pH ranging from 4.06 to 9.13 on the removal of MO are illustrated in Fig 4c. A downward trend on the removal efficiency was observed with increasing starting pH values. 98.1% of dye removal was obtained at the optimal initial pH 4.06 within approximately 10 minutes, where the observed rate constant approached 1.256 min^-1^ (S1 Table). The MO solution was decolorized to more than 91.2% when pH was changed from 4.97 to 7.96. To our knowledge, the MO is negatively charged and exhibits alkalinity when the solution pH is higher than 4.4. The pH in the range of 4.06 to 7.96 was favorable for the positively charged iron nanoparticles (< pH_zpc_ ≈ 8) adsorbed the negatively charged MO molecules. In addition, it is well known that pH value played a significant role in reducing contaminants by Fe^0^. An acid solution was favorable for iron to optimize the use of its reactive sites and hinder ferrous hydroxide precipitation on iron surfaces [44]. Elevating pH to 9.13, however, gave a poor removal efficiency of 78.8%. Whereas, the removal efficiency was still superior to those obtained from the previous researches [28,45] considering that the amount of Fe^0^ used in the experiment was very limited relative to the concentrations of the target pollutants. It is deduced that the adsorbed hydrogen ions on the biochar desorbed into solution and was neutralized during the course of the reaction, which could withstand the adverse effect of alkaline conditions. The effects of three anions (Cl^-^, SO4^2-^, and NO^3-^) in MO solutions treated by nZVI/BC_5_ are illustrated in Fig 4d. The results indicated that the removal efficiency in the control experiment (MO solution only decolorized by nZVI/BC_5_) was 91.9% and the presence of Cl^-^ almost had almost no influence on the ultimate removal efficiency. The reduced rate indicated that the chloride ion may compete with MO molecules to adsorb onto the iron surfaces. However, both sulfate and nitrate had adverse effects on the removal of MO. Their decolorization efficiencies of 81.9% and 64.5% were observed. The practical dye wastewater consists of some common anions such as chloride ion, sulfate, and nitrate, which could react with the iron particles or occupy the reactive sties on the surface of Fe^0^ [46]. Su and Puls reported that the oxyanions were expected to perform either sorption-dominated reactions (SO_4_^2-^) or reduction-dominated reactions (NO_3_^-^) with nZVI [47]. Therefore, it is necessary to take into account excess consumption of nZVI/BC composites by anions in dye wastewater treatment.

### Long-term effect of nZVI/BC

**Fig 5:**
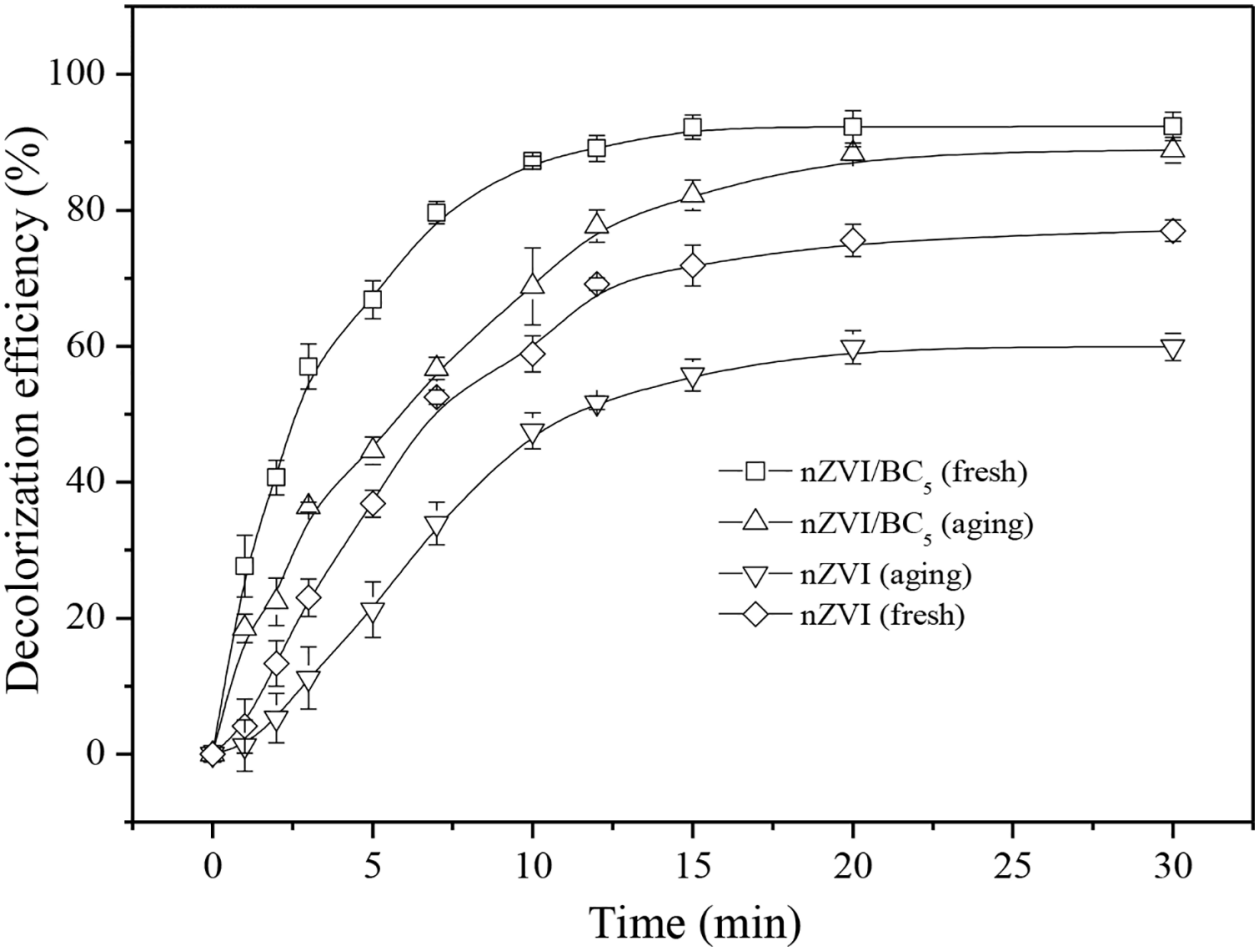
Effects of air aging of nZVI/BC_5_ on decolorization of MO, MO concentration = 300 mg/L, nZVI/BC_5_ dosage: = 0.6 g/L.

The effect of air aging materials on MO removal is shown in Fig 5. The fresh nZVI could remove 79.0% of 300 mg/L MO, whereas the decolorization efficiency by aged nZVI was decreased by 15%. The oxygen could convert Fe^0^ to ferrous or ferric oxide leading to a passivation layer forming on the nZVI surface. The Fe-Fe_x_O_y_ core-shell construction would restrain Fe^0^ in the core to react with contaminants [48]. As illustrated in Fig 5, the removal efficiency for aged nZVI/BC_5_ for a month was dropped only slightly by 3% in comparison with that of fresh nZVI/BC_5_ (92.3%). Therefore, nZVI modified by biochar seemed to be able to delay the formation of the passivated layer. The similar results were also obtained by other researchers using silicate clay mineral as supports [42]. Uzum et al. found that the oxide-layer thickness of iron nanoparticles supported by kaolinite clay aged for 8 months under normal conditions was no more than 5 nm [39]. The possible causes may be that the iron nanoparticles were synthesized in the pores of biochar or in the inner carrier surface, namely biochar may mechanistically prevent nZVI from interacting with air in the presence of biochar so that the oxidation formation was delayed [16,39].

### The mechanism of MO removal by nZVI/BC

**Fig 6:**
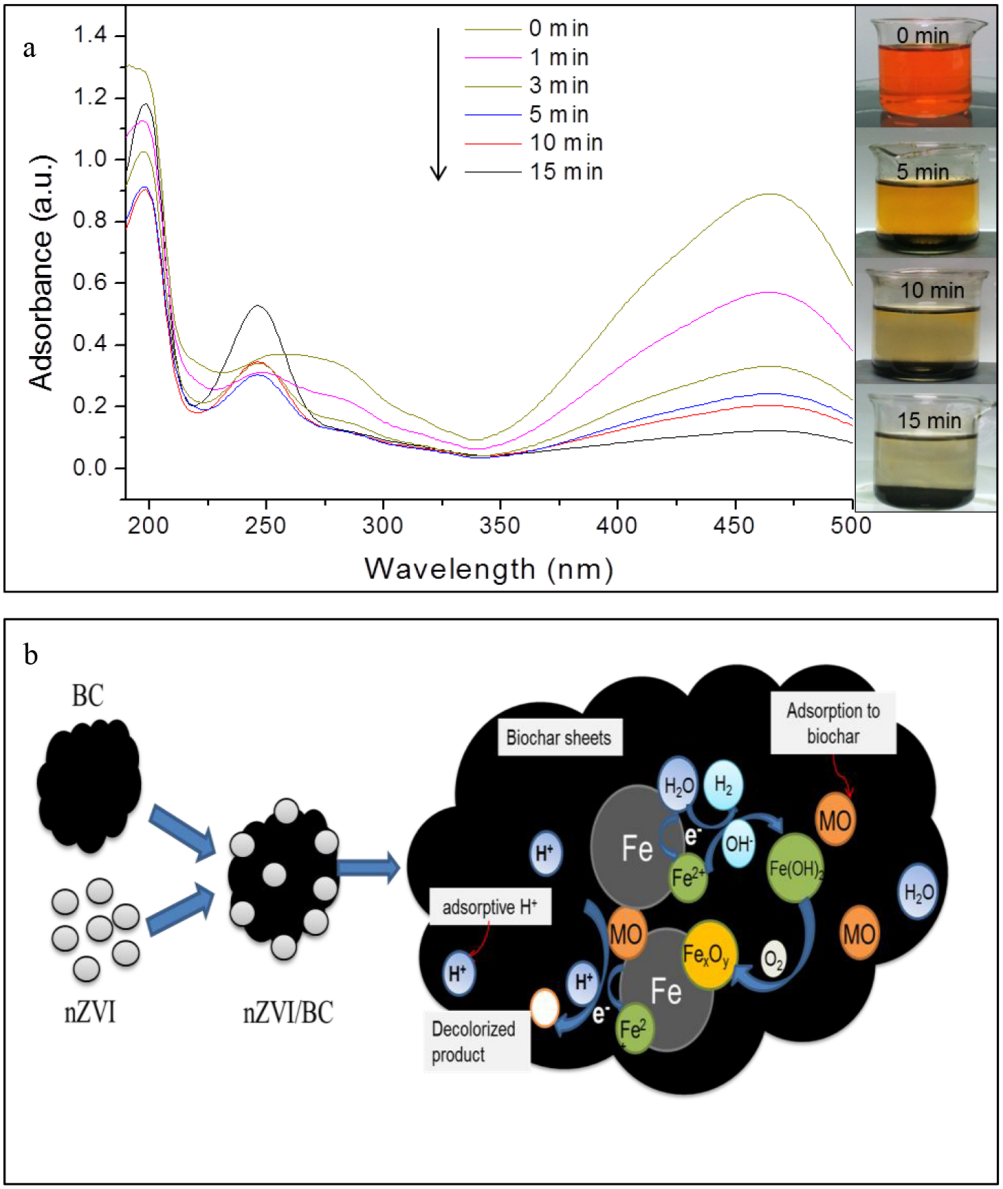
(a) UV-visible spectra of MO during the decolorization process and picture of colour variation of MO, Conditions: MO concentration = 300 mg/L, nZVI/BC_5_ dosage = 0.6 g/L; (b) The conceptual model for the removal mechanism of MO by nZVI/BC.

The UV-visible spectrum of MO decolorized by nZVI/BC_5_ and the visual representation with time are presented in Fig 6a. The color declined significantly in the first 5 min which was in line with the changing trend of UV-visible spectra. Subsequently, the color was almost disappeared within the 15 min. MO is an anionic dye and has a mono-azo group leading to its visible color. The chromophore group exhibited the maximum visible absorption wavelength at 464 nm and the π→π* transition related to aromatic rings caused absorbances at 270 and 191 nm, respectively [28,49]. As shown in Fig 6a, absorbance for the bands at 464, 270, and 191 nm was weakened gradually with the reaction time, indicating that the conjugated structure of MO was either destroyed or adsorbed by nZVI/BC_5_. In the meantime, the new peak of absorption at 248 nm in accordance with the UV-visible spectra of the standard solution of the sulfonic acid appeared and was enhanced with time [28], indicating that sulfonic acid was one of products and decolorization mechanism was mainly reduction.

On the basis of the collected experimental data, the conceptual model for the removal mechanism by nZVI/BC is illustrated in Fig 6b. Biochar-based nZVI was expected to play significant roles on both reduction and adsorption in the MO decolorization progress. Firstly, MO molecules were adsorbed on surfaces of biochar and nZVI due to their rich porosity, huge specific surface area, and π-π interactions between MO molecules and graphene layers of biochar [36]. Secondly, nZVI could transform the MO molecules by breaking down azo bond (-N = N-) to smaller molecular fragments including the sulfanilic acid and aromatic amine [29]. According to the degradation function of MO by nZVI (Fig 3), the reduction process mainly has two steps with the first step being reversible. In the first step, Fe^0^ supplied electrons to MO and combined H^+^ to form unstable intermediates, which could also return to the original material. In the second step, collisions between iron and MO occured and H^+^ was consumed further, resulting in the formation of aromatic amine and sulfonic acid in the second step [49]. Moreover, spontaneous reaction occurred between Fe^0^ and water to produce Fe^2+^ and H_2_ (Eq (4)). Hydrogen ions were used up vigorously during the reaction system resulting in the rise of the solution pH. Oxygen could transform Fe^0^ to iron oxides and decline the reducing capacity of iron nanoparticles. However, the porosity of biochar appeared to provide the protective layer for the nZVI and prevent iron nanoparticles from rapid oxidation and the mechanism needs to be investigated further.

## Conclusion

An environmental remediation composite of nZVI/BC for efficiently adsorbing and degrading organic pollutants could be synthesized using biochar supported iron nanoparticles. As characterized by TEM, XRD and BET analyses, biochar was capable of preventing the aggregation of iron particles and increasing the opportunities of contaminant interaction with nZVI. The increase in the mass ratio of biochar within the nZVI/BC composite was favorable for iron nanoparticles dispersing on biochar, whereas excess of biochar may block the reactive sites on the iron surface leading to the loss of the reducing capacity of irons. The mass ratio between biochar and nZVI at 5:1 appeared to give the best performance with the MO decolorization efficiency of 98.5%. Acid-washed biochar may supply more H^+^ to the oxidation-reduction reaction between Fe^0^ and MO molecules and maintain pH at neutral conditions. The effectiveness of the nZVI/BC for removal of MO was confirmed through a series of batch experiments under different initial conditions. Moreover, the removal efficiency for the aging nZVI/BC stored at neutral conditions for a month was slightly decreased, indicating that biochar supported nZVI could maintain long-term effectiveness for remediation of organic pollutants in environmental media.

Considering that biochar can be readily available, along with its cost-effectiveness, it may be a promising matrix to effectively decrease the aggregation of nZVI particles. The dispersed nZVI particles can strongly decolorize the dye-containing wastewater and the magnetic property of nZVI making the residues favorable for recollection by a magnet in aqueous solution. Therefore, the composite of nZVI/BC could offer an effective functional material for treating wastewater containing organic dyes.

## Supporting Information

S1 Table Parameters of non-linear equations for the empirical equation. (DOCX)
