# Supplementary material for: Biochar Supported Nanoscale Iron Particles for the Efficient Removal of Methyl Orange Dye in Aqueous Solutions
Source: PLoS One. 2015 Jul 23;10(7):e0132067. doi: 10.1371/journal.pone.0132067 (PMC4512678; doi:10.1371/journal.pone.0132067)
Supplement: S1 Table — (DOCX) [file pone.0132067.s001.docx]

**Supplementary Information**

Biochar supported nanoscale iron particles for the efficient removal of methyl orange dye in aqueous solutions

Lu Han^1¶^, Song Xue^1, 2¶^ , Shichen Zhao^3^, Jingchun Yan^1^, Linbo Qian^1^, Mengfang Chen^1*^

^1^ Key Laboratory of Soil Environment and Pollution Remediation, Institute of Soil Science, Chinese Academy of Sciences, Nanjing 210008, China.

^2^ College of Environmental Science and Engineering, Suzhou University of Science and Technology, Suzhou 215011, China

^3^ College of Resource and Environmental Sciences, Nanjing Agricultural University, Nanjing 210095, China

* Corresponding author

Tel.: +86-25-86881847; fax: +86-25-86881126.

E-mail address: mfchen@issas.ac.cn.

^¶^ These authors contributed equally to this work.

Table S1. Parameters of non-linear equations for the empirical equation.

| Material | C_0_ (mg/L) | Additional dosage (g/L) | Initial pH | Anions | Empirical equation | | | |
| --- | --- | --- | --- | --- | --- | --- | --- | --- |
|  |  |  |  |  | C_e_ (mg/L) | α | k min^-1^ | R^2^ |
| nZVI | 60 | 0.1 | 5.76 | / | 4.235 | 1.058 | 0.104 | 0.979 |
| nZVI/BC_3_ | 60 | 0.4 | 5.64 | / | 7.476 | 0.975 | 0.561 | 0.971 |
| nZVI/BC_5_ | 60 | 0.6 | 5.67 | / | 5.422 | 0.986 | 0.743 | 0.963 |
| nZVI/BC_7_ | 60 | 0.7 | 5.71 | / | 6.213 | 0.984 | 0.476 | 0.972 |
| nZVI/BC_5_ | 200 | 0.6 | - | / | 22.668 | 0.958 | 0.470 | 0.974 |
| nZVI/BC_5_ | 400 | 0.6 | - | / | 53.210 | 0.961 | 0.319 | 0.982 |
| nZVI/BC_5_ | 600 | 0.6 | - | / | 163.635 | 1.005 | 0.303 | 0.992 |
| nZVI/BC_5_ | 300 | 0.3 | - | / | 161.090 | 1.093 | 0.201 | 0.972 |
| nZVI/BC_5_ | 300 | 0.6 | - | / | 31.126 | 0.994 | 0.298 | 0.989 |
| nZVI/BC_5_ | 300 | 0.9 | - | / | 19.989 | 1.012 | 0.369 | 0.975 |
| nZVI/BC_5_ | 300 | 0.6 | 4.06 | / | 12.961 | 0.994 | 1.256 | 0.989 |
| nZVI/BC_5_ | 300 | 0.6 | 4.97 | / | 35.888 | 0.956 | 0.663 | 0.934 |
| nZVI/BC_5_ | 300 | 0.6 | 7.96 | / | 17.288 | 0.989 | 0.126 | 0.991 |
| nZVI/BC_5_ | 300 | 0.6 | 9.13 | / | 64.135 | 1.027 | 0.271 | 0.980 |
| nZVI/BC_5_ | 300 | 0.6 | - | NO_3_^-^ | 74.089 | 1.090 | 0.142 | 0.977 |
| nZVI/BC_5_ | 300 | 0.6 | - | SO_4_^2-^ | 33.317 | 1.007 | 0.155 | 0.993 |
| nZVI/BC_5_ | 300 | 0.6 | - | Cl^-^ | 27.700 | 0.952 | 0.133 | 0.957 |

Note: - denote not determined; / denote null
